# Deep scaffold hopping with multimodal transformer neural networks

**DOI:** 10.1186/s13321-021-00565-5

**Published:** 2021-11-13

**Authors:** Shuangjia Zheng, Zengrong Lei, Haitao Ai, Hongming Chen, Daiguo Deng, Yuedong Yang

**Affiliations:** 1 School of Data and Computer Science, Sun Yat-Sen University, China, 132 East Circle at University City, Guangzhou, 510006 China; 2 Fermion Technology Co., Ltd, 1088 Newport East Road, Guangzhou, 510335 China; 3 Centre of Chemistry and Chemical Biology, Guangzhou Regenerative Medicine and Health Guangdong Laboratory, Guangzhou, 510530 China

**Keywords:** Deep learning, Drug design, Scaffold hopping, Molecular optimization, Transformer neural network

## Abstract

Scaffold hopping is a central task of modern medicinal chemistry for rational drug design, which aims to design molecules of novel scaffolds sharing similar target biological activities toward known hit molecules. Traditionally, scaffolding hopping depends on searching databases of available compounds that can't exploit vast chemical space. In this study, we have re-formulated this task as a *supervised molecule-to-molecule translation* to generate hopped molecules novel in 2D structure but similar in 3D structure, as inspired by the fact that candidate compounds bind with their targets through 3D conformations. To efficiently train the model, we curated over 50 thousand pairs of molecules with increased bioactivity, similar 3D structure, but different 2D structure from public bioactivity database, which spanned 40 kinases commonly investigated by medicinal chemists. Moreover, we have designed a multimodal molecular transformer architecture by integrating molecular 3D conformer through a spatial graph neural network and protein sequence information through Transformer. The trained DeepHop model was shown able to generate around 70% molecules having improved bioactivity together with high 3D similarity but low 2D scaffold similarity to the template molecules. This ratio was 1.9 times higher than other state-of-the-art deep learning methods and rule- and virtual screening-based methods. Furthermore, we demonstrated that the model could generalize to new target proteins through fine-tuning with a small set of active compounds. Case studies have also shown the advantages and usefulness of DeepHop in practical scaffold hopping scenarios.

**Supplementary Information:**

The online version contains supplementary material available at 10.1186/s13321-021-00565-5.

## Introduction

Over the past decades, the hit identification process of drug discovery has been largely facilitated by the rapid developments of both high-throughput screening (HTS) and fragment-based screening technologies [1]. These screening strategies, together with the combinatorial compound library, discover extensive collections of diverse chemical series. Though these identified compounds usually have weak potency and do not necessarily possess an ideal ADMET profile, they are starting points (hits) to identify more potent lead compounds through lead optimization or lead identification. [2]

One common strategy in the lead optimization is the scaffold hopping coined by Schneider and co-workers [3], where a given reference compound was modified in the backbone to generate structurally distinct compounds while keeping the three-dimensional shape or the pharmacophore in order to preserve the biological activity against its target protein [3, 4]. The strategy has been widely used because such design can result in novel chemotypes that have improved properties and/or achieve intellectual property rights. However, "hop" to a hit molecule is not guaranteed to work in an expected way due to an incomplete understanding of the protein–ligand interaction mechanism [5], unfavorable ADMET properties of the hopped structure, or activity cliff [6]. Empirical scaffold transformation rules like ring-break/opening or bioisostere theory [7] summarized by medicinal chemists are insufficient to overcome the sophisticated real-world cases.

To help chemists find better scaffold hops, there are a variety of computational methods having been proposed, including 3D shape-based similarity search, fingerprint-based similarity search, pharmacophore matching, and fragment replacement techniques [4, 8–19]. These methods mainly relied on a predefined database to select a molecule or a fragment, with the differences between approaches arising from the searching algorithms of the database, the ways to define the similarity of compound pairs or the contents of the scanned database. Notwithstanding the solid performance of existing scaffold hopping methods, there remain three main challenges. First, the number of potential hops for a chemotype is too large to be memorized and requires considerable creativity and experience. As an example, the widely-used virtual VEHICLe database [20] has 24,847 scaffolds, but it is composed of almost heteroaromatic mono- and bicycles. It remains intricate to maintain a balance between diversity, size, and computational cost. Secondly, most of the currently utilized molecular fingerprints are the result of algorithms involving some degree of knowledge-guided or manual feature engineering. While these representations can clearly be successful, they always feature a trade-off in assigning importance to certain molecular features while neglecting others, with this choice hand-coded in the algorithm and not amenable to problem-specific tuning [21]. Lastly, these methods depend on a predefined database and can't cover the vast chemical space estimated to contain 10 [23] and 10 [60] drug-like molecules [22]. Therefore, it's necessary to develop novel schemes that can automatically dig into the prioritized chemical space while providing bespoke molecular representation for hops identification.

In parallel, upon call for a more exhaustive and intelligent exploration of chemical space, the de novo molecule design has been advanced by recent breakthroughs in deep generative models [23, 24]. Various generative architectures, including RNNs [25–27], autoencoders [28, 29], and generative adversarial networks (GANs) [30] have been proven effective for generating desirable molecules by representing molecules with either the simplified molecular input line entry specification (SMILES) [31] or molecular graph [32]. Recent works also provide alternatives by combining the reinforcement learning and docking methods to generate compounds that satisfy key residue interactions with target protein [33–35]. These methods aimed to design structurally diverse compounds from scratch and thus have the capability to search the whole drug-like space without relying on any predefined database or rules. Albeit powerful, these de novo design approaches are rarely consider molecular optimization based on existing reference compounds.

Based on these observations, two research lines were recently carried out for molecule design under scaffold constraints. The first research line is called scaffold-based molecule design proposed by Lim et al. [36] and Li et al. [37], where the graph generative models were utilized to extend a given scaffold by sequentially adding atoms and bonds. In this context, the generated derivatives are guaranteed to maintain the scaffold with certainty, and their properties can thus be controlled by conditioning the generation process on desired properties. However, the generated molecules often differ significantly from the starting points in the 3D level, and many of the proposed transformations are R-group modifications [38]. The other line is referred to as fragment linking first proposed by Imrie and co-workers, where the original idea is to join fragments together with a generated linker while keeping the relative conformations of the fragments [39]. Yang et al. further extended it as a sentence completion problem through transformer neural networks [40]. Although these approaches claim their capability in scaffold hopping to generate molecules with high 3D similarities to the original molecule, their generated molecules often have higher 2D similarities than expected due to the nature of fragment replacement, resulting in unfavorable intellectual property issues. Moreover, all these models were trained in a ligand-based paradigm using a large number of bioactive compounds from the different public databases without using the information of the specific target proteins, imposing a limit in applications into the target-centric drug development process.

In this study, for the first time, we re-formulate the scaffold hopping task as a *supervised molecule-to-molecule translation* instead of search problem. Given a reference molecule and a specified protein target, our goal is to design scaffold hops incorporating 2D and 3D structural information, protein target information, as well as bioactivity information. To this end, we have developed a novel target-based scaffold hopping framework, DeepHop, to optimize hit/lead compounds based on a multimodal deep generative model. The model has been trained with over 50 K constructed scaffold hopping pairs across 40 kinases. Extensive experiments show that our model is capable of generating isofunctional molecular structures for seed molecules with novel backbones and improved activity. More importantly, our model could be easily extended to new protein targets outside the training set, which is essential for target-centric drug development.

## Methods

### Task definition

**Fig. 1:**
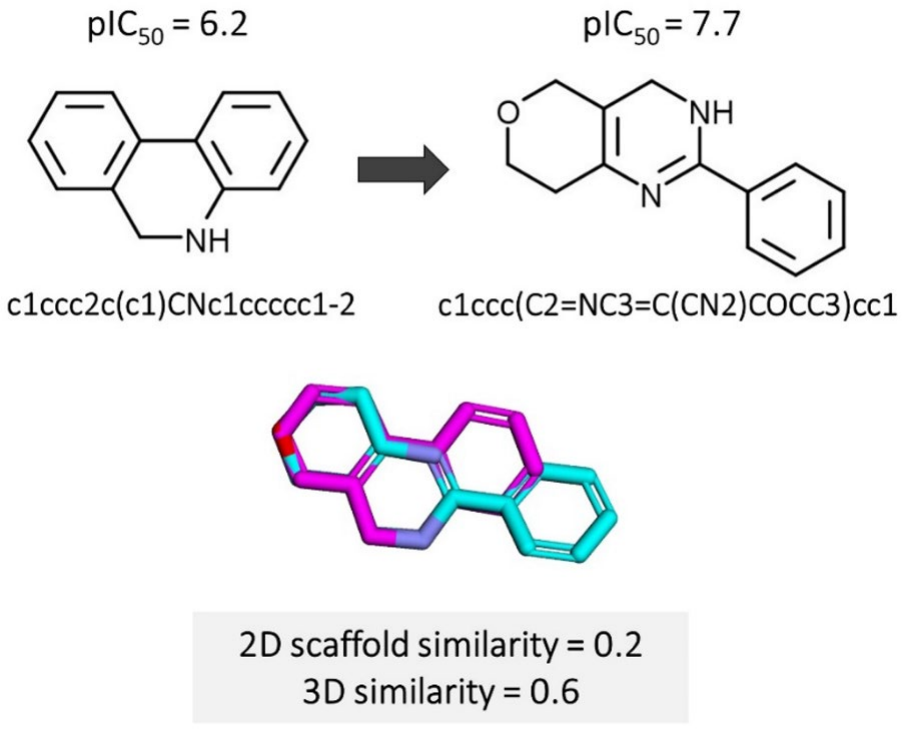
A typical scaffold hop extracted from tankyrase-2 inhibitors [4]. The two compounds have improved bioactivity (pIC_50_ increase of 1.5) and similar 3D shapes (3D shape and pharmacophore similarity = 0.6) but different scaffolds (2D Tanimoto scaffold similarity = 0.2)

An exemplary scaffold hop is shown in Fig. 1. In this work, we broadly define a scaffold hopping process as such: given an input reference molecule X and a specified protein target Z, the model predicts the "hopped" molecule Y with the improved pharmaceutical activity and similar 3D structure but dissimilar 2D structure.

### Data preparation

There have only been a limited number of successfully reported examples for scaffold hopping. As a proof of concept, we constructed sets of scaffold-hopping pairs using a custom-made similarity scoring function from a subset of ChEMBL20 [41].

Specifically, we processed the ChEMBL20 dataset by filtering kinase-related target proteins with at least 300 up to 5000 unique bioactivity instances. The scaffold hopping application in the kinase family has always been a topic of interest because the kinase patent literature is notoriously complicated and hard to break [42]. We further filtered out the SMILES strings containing disconnected ions or fragments. The molecules were then normalized using RDKit, which involved the removal of salt and isotopes, as well as charge neutralization. After the preprocessing, the final data set contained 103,511 bioactivity data points across 152 kinases. Note that we used pChEMBL values as the standard activity unit, which were defined as: -Log(molar IC_50_, Ki, and Kd).

### Deep QSAR model

Before constructing the scaffold hopping pairs, one important factor required to assess the performance of scaffold hopping is whether the generated molecules have similar bioactivity on the desired targets. To enable a rapid and accurate profiling of generated molecules, virtual profiling models were trained on all the data points in the whole kinase datasets. We evaluated the state-of-the-art directed messages passing neural networks (DMPNN) [43] and multi-task deep neural networks (MTDNN) [44] with molecular graphs or molecular fingerprints as the molecular representations. In particular, MTDNN was found to obviously outperform DMPNN with an average R^2^ of 0.62 and RMSE of 0.61 (pCHEMBL value) on internal test sets. Thus, the MTDNN model was used as the virtual profiling model in the following studies. The modeling details and results are shown in Supplementary files. For the quality of the virtual bioactive assessments, we kept only targets that had a fivefold cross-validation R^2^ higher than 0.70, resulting in 40 targets in the end.

### Construction of scaffold hopping pairs

The scaffold hopping definition emphasized two key components: (i) different core structure and (ii) similar topology and pharmacophore that ensure improved biological activities of the new compounds relative to the parent compounds. To mimic the scaffold hopping scenario, we constructed our data set following the idea of matched molecular pairs (MMPs) proposed by Hussain et al. [45]. More specifically, we sampled target-based hopping pair ((X; Y)|Z) with a significant bioactivity improvement (pCHEMBL Value ≥ 1) for new compound Y over original compound X in the context of protein Z and a strict molecular similarity condition (2D scaffold similarity (X; Y) ≤ 0.6) ∩ (3D similarity (X; Y) ≥ 0.6). Following the recent study by Imrie et al [39], we measured 2D scaffold similarity through the Tanimoto score over Morgan fingerprints [46] of the compound scaffolds (here referred specifically to the Bemis and Murcko (BM) scaffold [47]), and 3D molecular similarity through the shape and color similarity score (SC score) (the pharmacophoric feature similarity [48] and the shape similarity [49]). To compute the SC score, we sampled 100 conformations for each molecule using RDKit MMFF94 force filed and selected only the lowest-energy conformation. The SC score is a float value in the range of [0, 1], with a higher value representing a higher similarity between molecule pairs. Scores above 0.6 indicate fair structural matches, and those above 0.8 indicate an excellent match.

To avoid redundancy of training pairs, we only allowed up to 10 hops for each source molecule. For each target, we first randomly selected 10% bioactive molecules as the test set and used the rest 90% molecules to construct scaffold hopping pairs for training and validation by a ratio of 9:1. These processing steps resulted in a training set of 57,537 pairs and a test set of 3656 molecules over 40 kinases.

It should be noted that our method could be adapted to other applications by simply constructing molecular training pairs satisfying the specific requirements. We employed such a definition of scaffold hopping since there is no generally preferred definition of core structures or scaffolds or accepted metrics available for evaluating the scaffold hopping potential.

### Independent test set

To explore the generalization ability of proteins that have never been observed during the training process, we retrieved six targets from the rest of the curated database as the independent test set. Among them, three proteins (CHEMBL2208, CHEMBL2147, CHEMBL2523) are non-homologous with sequence identity less than 25% (calculated by the CDhit [50]) to any sequence in the training set, while others (CHEMBL4225, CHEMBL2292, CHEMBL2041) are homology to the training set with the highest sequence identities of 59, 63, 76%, respectively. The compounds in these six proteins have never be observed in the model training, validating, and testing processes. The details of these six proteins are shown in Additional file 1: Table S3.

### Model architecture

A novel multimodal graph transformer model was proposed for generating scaffold hops with inputs of a source molecule and a protein sequence based on the transformer architecture [51]. As classical encoder-decoder architecture, Transformer has recently shown the state of the art performances in many sequence-to-sequence translation tasks, including machine translation [52], retrosynthesis [53], and fragment assembly [40]. In previous chemical applications like retrosynthesis and fragment assembly, chemical structures were often converted into SMILES strings that ignored spatial information naturally embedded in chemical 3D conformers. Also, none of them considered the protein target information during the transformation of the molecule pairs. Obviously, both of these two features play crucial roles in the scaffold hopping task that needs to be considered.

**Fig. 2:**
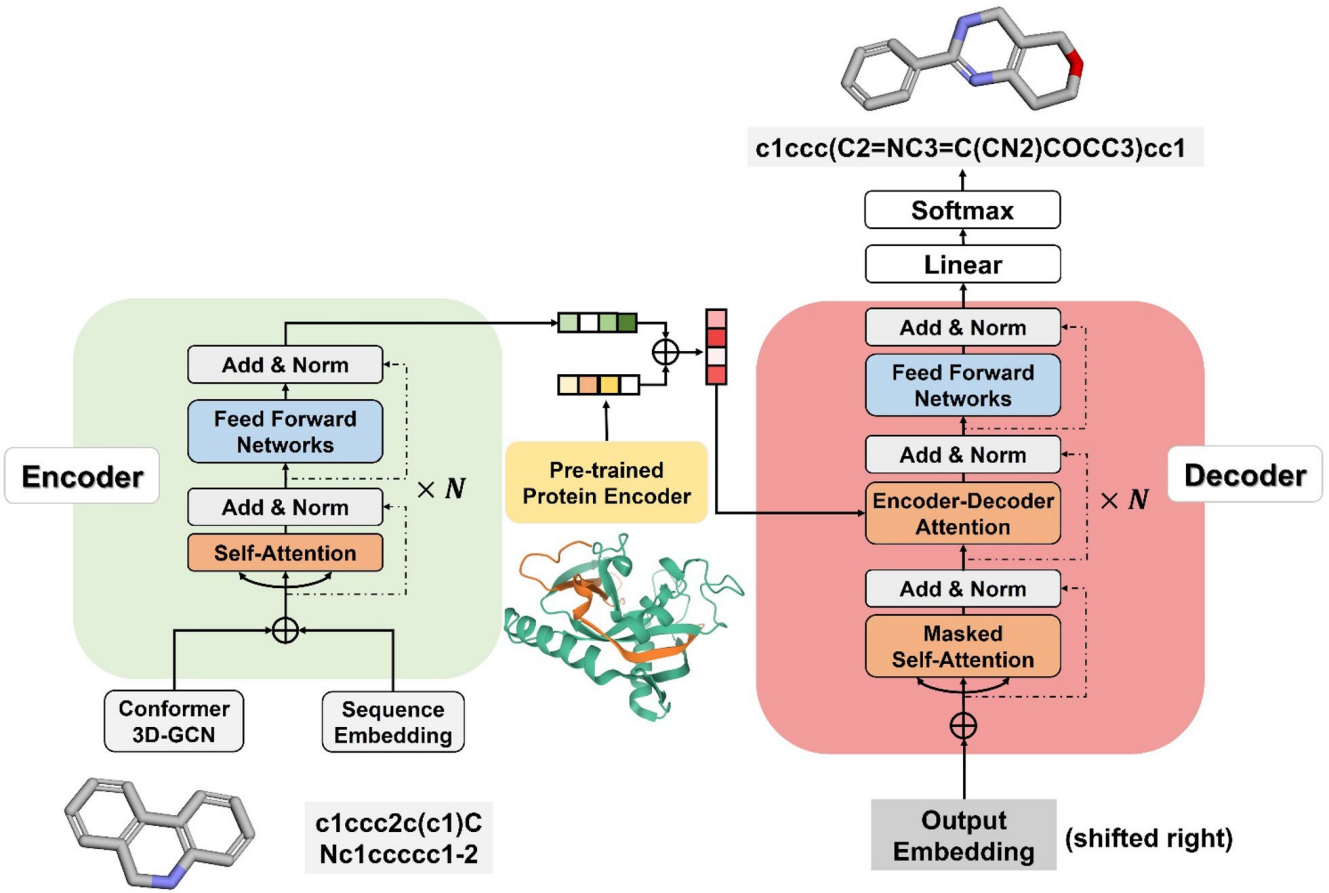
The basic architecture of the multimodal transformer model DeepHop. The model comprises three main components: (1) a 3D graph neural network for molecular conformer embedding, (2) a pre-trained encoder for the target protein embedding, and (3) a transformer for mapping the scaffold hopping pairs

As shown in Fig. 2, DeepHop comprises three main components: (1) a molecular 3D graph neural network (GNN) for molecular conformer embedding, (2) a pre-trained encoder for target protein embedding, and (3) a transformer for mapping the scaffold hopping pairs.

### Molecular 3D conformer encoder

We adopted a simple 3D spatial GNN as the molecular conformer encoder following the strategy of Danel et al. [54], which can learn both the molecular graph representation and spatial distances between atoms in the 3D space. The GNN follows the paradigm of message passing neural networks. The input of the conformer encoder is a 3D molecular graph $G=(V, E)$, where $V=\left\{ {v}_{1},\dots ,{v}_{n}\right\}$ denotes a set of nodes (atoms) and $E={[{e}_{ij}]}_{i,j=1}^{n}$ represents edges (bonds) between atoms *i* and *j*. Each atom ${v}_{i}$ is represented by a *d*-dimensional initial feature vector *h*_*i*_ containing the 2D chemical features computed by RDkit (See more details in Additional file 1: Table S1). The atom is additionally attached with its 3D coordinates ${p}_{i} \in {\mathbb{R}}^{3}$ obtained by the molecular conformer. The 3D GNN then updates the atom embedding with message passing operations:

$$h_{i}^{{\left( {l + 1} \right)}} (U,b) = \sum\limits_{{j \in N_{i} }} R eLU\left( {(U^{T} (p_{j} - p_{i} ) + b) \odot h_{j}^{\left( l \right)} } \right)$$

where ${h}_{j}^{(l)}$ is *d-*dimension the feature vector of atom (node) *j* at the *l-*th updating iteration, ${N}_{i}$ is the set of neighbored atoms to atom *i*, $U\in {\mathbb{R}}^{t\times d}$ and $b\in {\mathbb{R}}^{d}$ are trainable network parameters and $\odot$ denotes element-wise multiplication.

Herein, the overall atom embeddings of the molecule (graph) can be described as ${H}^{(l)}=\{{h}_{1}^{(l)},\dots {h}_{n}^{(l)}\}$. In the last iteration of the node embedding updating, inspired by a recent molecular representation model [43], we introduced a Gated Recurrent Unit (GRU) network [55] to increase the power of the network and obtained the final atom embeddings, as shown as

$$\widehat{H}\left(v\right)=GRU({H}^{(l)}\left(v\right))$$

where ${H}^{(l)}\left(v\right)$ is the set of atom representations in the molecular graph $G$.

### Protein encoder

Compared to the drug molecules, protein molecules are much bigger, typically containing more than 1,000 heavy atoms. To avoid a bulky model that contains too many parameters, we adopted the Tasks Assessing Protein Embeddings (TAPE) [56], a recently proposed semi-supervised protein sequence representation learning method, to generate the protein pre-trained embeddings. TAPE was trained by a large transformer neural network in an unsupervised paradigm with millions of protein sequences. After training, it can generate an information-enriched feature vector for an input protein sequence. Formally, a protein can be described as a linear sequence that consists of a list of amino acid residues $P=({r}_{1},\dots {r}_{l})$. After processing through the TAPE, a vector ${H}_{p}$ can be obtained as a *k*-dimensional pre-trained feature vector.

### Transformer architecture

The fundamental architecture of DeepHop is a typical Transformer neural network containing multiple encoder-decoder modules. Each encoder layer consists of a multi-head self-attention sub-layer and a position-wise feed-forward network (FFN) sub-layer. Multi-head attention has several scaled dot-product attention functions working in parallel, which allows the model to focus on messages from different subspaces at different positions. The attention between query (Q), keys (K), and values (V) was computed as

$$Attention\left(Q, K, V\right)=softmax\left(\frac{{QK}^{T}}{\sqrt{{d}_{k}}}\right)V,$$

where a scaling factor ${d}_{k}$ (equal to the size of weight matrices) was introduced to avoid excessive dot products. The FFN sub-layer adopts the ReLU activation [57]. Then, layer normalization [58, 59] and a residual connection [60] were introduced to link the above two sub-layers. Each decoder layer has three sub-layers, including an FFN sub-layer and two attention sub-layers. The decoder self-attention sub-layer utilizes a mask function to hinder attending to unseen future tokens. The encoder-decoder attention layer helps the decoder to focus on essential parts in the source sequence, and to capture the relationship between the encoder and decoder.

For a given source molecule, we concatenate the learned 3D graph representations $\widehat{H}\left(v\right)$ with SMILES sequence embedding *M*_*s*_ = (*s*_*1*_*, …, s*_*m*_) in atomic level and convert them through a simple linear transformation. The combined multimodal molecular representations are then sent to the Transformer encoder to convert into a latent representation $L\in {\mathbb{R}}^{m\times f}$, where *m* is the sequence length of molecular SMILES and *f* is the hidden state dimension. Afterward, we concatenate $L$ with target protein embedding ${H}_{p}\in {\mathbb{R}}^{k}$, resulting in a comprehensive representation $\widehat{L}\in {\mathbb{R}}^{m\times (f+k)}$. Given $\widehat{L}$, the decoder iteratively generates an output SMILES sequence *Y* = (*y*_*1*_*, …, y*_*o*_) until the ending token "⟨/s⟩" is generated.

During training, the model minimizes the cross-entropy loss between the target sequence *M*_*t*_ = (*t*_*1*_*, …, t*_*k*_) and the output sequence *Y*.

$$\mathcal{L}\left(Y, M\right)=-\sum_{i=1}^{k}{y}_{i}\mathit{log}{t}_{i}$$

### Baseline models

We compare our approaches with the following baselines:

#### Conventional methods

1. **Ligand-based virtual screening (LBVS).** Here, we prepared a ZINC lead-like compound library by following the strategy of Moses [60], containing 1,936,963 molecules with 448,854 unique Bemis-Murcko scaffolds. For a fair comparison, we randomly selected 50,000 molecules in the library (equaling to our training set size) and chose top-10 molecules with the highest 3D similarity to the reference molecule as the final hops. The molecules with 2D-similarity higher than 0.6 were pre-excluded from the random selection in the library.
2. **MMPA**. MMPA was performed by the implementation by Hussain et al. [45], where molecular transformation rules were extracted from the kinase dataset for corresponding tasks. During the test, we translated a source molecule 10 times using different matching transformation rules and selected the top-10 translations with the highest average bioactivity as scored by the virtual profiling model if there are more than 10 matching rules.

#### Deep learning methods

3. **Seq2seq**. The seq2seq model utilizes SMILES strings to encode molecules. It consists of an LSTM encoder and an LSTM decoder with an attention mechanism. This architecture has been successfully applied to other molecular *de novo* design and molecule transformation tasks [61].
4. **G2G**. The fourth baseline is a Graph-to-Graph model [62] that extends the junction variational autoencoder (VAE) via attention mechanism and generative adversarial networks (GAN). The model is capable of translating the current molecule to a similar molecule with predefined desired property (e.g., logP).

Notably, these algorithms were not designed for multi-task transformation. We randomly chose four targets as representatives to evaluate the effectiveness of the baselines and our model.

### Evaluation metrics

The scaffold hopping methods is often not comparable, similar to many virtual screening studies, partly due to the inconsistent definition of scaffold hop and lack of accepted benchmarks. We quantitatively analyze the hopping success rate, bioactivity improvement, validity, uniqueness, diversity, and novelty of different methods.

- **Success rate** is a metric that considers both similarity and bioactivity improvement. Since this task aims to generate a molecule that (i) has a different scaffold from the input molecule and (ii) has bioactivity improvement simultaneously. We design criteria to judge whether it satisfies these two requirements by: the generated molecule Y should (a) meet the structural condition, i.e., (2D scaffold similarity (X; Y) ≤ 0.6)$\cap$(3D similarity (X; Y) ≥ 0.6); (b) has a positive bioactivity gain, i.e., pBioactivity(Y)—pBioactivity(X) ≥ 0, where the activity of generated molecules was computed through the deep QSAR models. A **constraint success rate** is also accounted for by confining a significant increase of bioactivity as: pBioactivity(Y)—pBioactivity(X) ≥ 1.
- **Bioactivity improvement** is the average improvement of biological activity between the source molecule and the generated molecule computed as pBioactivity(Y)—pBioactivity(X)
- **Validity** is the percentage of generated molecules that are chemically valid according to RDkit;
- **Uniqueness** refers to the number of unique structures generated;
- **Novelty** refers to the percentage of novel molecules (not present in the training set) among the chemically validly generated molecules.

### Model training and optimization of hyperparameters

The DeepHop model was implemented based on OpenNMT [63], and all scripts were written in Python [64] (version 3.7). The models were trained on four GPU (Nvidia 2080Ti) and saved checkpoint per epoch. The best hyperparameters were decided based on the loss of the validation set (See more details in Additional file 1: Table S2). We adopted the beam search procedure [65] to generate multiple candidates with different beam widths. All generated candidates were canonicalized using RDkit and compared to the source molecules.

## Results and discussion

In this section, we mainly discussed our DeepHop performance from four parts. First, we evaluated our model with different training paradigms on the whole dataset. Then, we compared our methods with the state-of-the-art deep learning models as well as conventional methods on four internal proteins. Subsequently, we tested our model in unseen protein sets and performed few-shot transfer learning on proteins with low performance. Lastly, our DeepHop method was applied to several case study examples to demonstrate the capability of the model for practical scaffold hopping.

### Evaluation of DeeopHop on the multi-kinase dataset

We first assessed the performance of methods on the internal test set with different training paradigms, including single-task, DeepHop-noGNN, DeepHop-noProtein, and DeepHop. The top 10 candidate sequences for each reference compound were generated. As shown in Table 1, by averaging on 40 targets, our multimodal DeepHops achieved the best overall performance with a success rate of 65.2 ± 17.5 and constraint success rate of 43.7 ± 21.0. By comparison, the single-task method, which has separately trained and evaluated 40 models for each target protein, achieved the worst performance in most of the metrics with a success rate = 27.5 ± 15.9, constraint success rate = 15.5 ± 14.7. Specifically, the average validity is only 12.9 ± 6.3, much lower than > 90% by all other three methods. These should be caused by the relatively small number of data points for each single kinase task, leading to a fragile model that is difficult to learn the transformation between scaffold pairs. When integrating all the pairs from different kinase sets for DeepHop-noProtein, the model can capture key structural information in molecular translation, achieving a success rate of 58.9% and a constraint success rate of 34.6. However, its average bioactivity improvement is 0.64, much lower than the 0.97 by DeepHop due to a lack of protein target information input to the model. On the other hand, DeepHop-noGNN, an removal of the 3D GNN module from DeepHop, also decreases the success rate by 3.4%, demonstrating the effectiveness of the 3D conformer information of input molecules. The separate results over targets are shown in Additional file 1: Figs. S1–S3.

**Table 1:** Performance comparison of different training settings by the average and standard deviation on the internal test set of 40 protein targets

Metrics	Models			
	Single-task	DeepHop-noGNN	DeepHop-noProtein	DeepHop
Success rate (%)	27.5_(15.9)_	61.8_(18.6)_	58.9_(20.9)_	**65.2**_(17.5)_
Constraint success (%)	15.5_(14.7)_	40.6_(20.9)_	34.6_(19.7)_	**43.7**_(21.0)_
Improvement	0.53_(0.31)_	0.92_(0.27)_	0.64_(0.28)_	**0.97**_(0.24)_
Validity (%)	12.9_(6.3)_	94.4_(2.7)_	92.7_(3.8)_	**95.7**_(3.8)_
Uniqueness (%)	8.7_(5.5)_	74.6_(11.1)_	**88.2**_(8.8)_	76.4_(9.3)_
Novelty (%)	99.0_(0.9)_	99.5_(0.5)_	**99.6**_(0.3)_	99.4_(0.5)_

The best performing numbers are in bold

The numbers in brackets are the standard deviation

### Performance comparison with other methods

We further compared DeepHop with baseline methods. Since other baseline methods need to re-train the model or screen the whole database that is slow to run, we randomly selected four protein targets for comparison. As shown in Table 2, DeepHop achieved the best average success rate (65.1%) and constraint success rate (33.5%), consistent with the previous benchmark over the whole dataset. The rates by DeepHop are both around two times higher than four baseline methods and are shown to consistently outperform other methods in four protein targets (Additional file 1: Tables S4–S7). In contrast, four baseline methods achieved similar success rates in the range of [29.1, 34.6] and constraint success rates of [10.4, 15.9]. Among these, two deep learning-based methods, Seq2seq and G2G, both suffer from a low uniqueness of 18%. In addition, Seq2seq has a very low validity of 29.2% due to the internal difficulty of generating valid SMILE texts. These should be caused by the limited number of training data on single target data because the half success rate is similar to the level achieved by DeepHop (Single-task) on the test set. DeepHop alleviates this issue by integrating 40 protein target data sets and thus achieve relatively stable results in different targets. On the other hand, in conventional methods, LBVS achieved the lowest constraint success rate of 10.4%. This is because LBVS could ensure 100% validity and uniqueness through the database search, but it is hard to find optimized hits due to the limit of available compounds with an average bioactivity improvement of − 0.95. The MMPA improves the constraint success rate by increasing the average bioactivity improvement of generated compounds with a slight loss in validity and uniqueness.

**Table 2:** Performance comparison of five methods on four internal protein targets

Metrics	Models				
	LBVS	MMPA	Seq2seq	G2G	DeepHop
Success Rate (%)	34.6_(16.4)_	33.9_(13.2)_	29.1_(16.6)_	33.4_(6.6)_	**65.1**_(12.7)_
Constraint Success (%)	10.4_(7.2)_	13.4_(6.1)_	15.9_(8.5)_	14.7_(1.5)_	**33.5**_(8.8)_
Improvement	− 0.94_(0.61)_	0.31_(0.15)_	0.77_(0.47)_	**0.88**_(0.65)_	0.81_(0.32)_
Validity (%)	–	97.3_(0.04)_	29.2_(0.11)_	**99.7**_(0.03)_	93.9_(0.02)_
Uniqueness (%)	–	**99.2**_(0.01)_	17.8_(0.03)_	17.9_(0.15)_	70.7_(0.05)_
Novelty (%)	–	**–**	99.1_(0.03)_	**100**_(0.00)_	99.5_(0.01)_

Reported are average and standard deviation (numbers in brackets) over six metrics

**Fig. 3:**
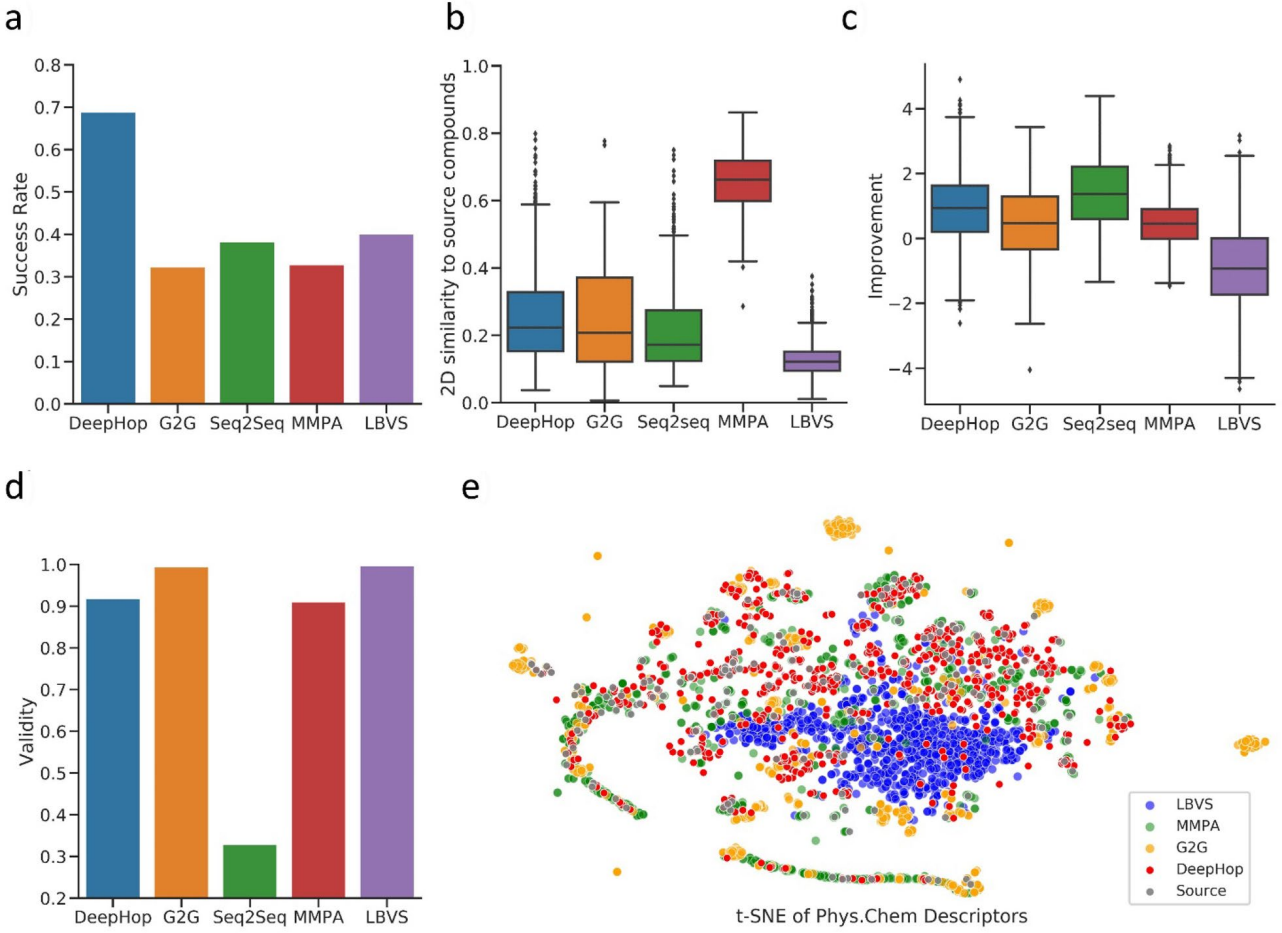
The statistical performance of different methods for CHEMBL267 over **a** success rate, **b** 2D similarity to source compounds, **c** bioactivity improvement, **d** validity. We also show **e** t-SNE projection of physicochemical descriptors of the source compounds and compounds generated by Deephop and several baseline models

We took CHEMBL267, which has a success rate close to the average of four targets, as a representative to analyze the performance. We conclude the statistical performance of different methods for CHEMBL267 over (a) success rate, (b) 2D similarity to source compounds, (c) bioactivity improvement, (d) validity. To compare the physicochemical properties of the compounds generated by various models, we first computed the 200 common physicochemical properties of each molecule by RDkit following Yang et al. [66]. We then implemented dimensionality reduction to 2D with t-SNE. As a result, we found that MMPA could generate property-aligned molecules (Fig. 3e) that are highly similar in 2D to original ones (Fig. 3b) and cannot provide novel scaffolds. This is unfavorable in scaffold hopping scenarios. LBVS, though generating compounds with the lowest 2D similarity on average, is hard to produce appropriate hops due to the decreased bioactivity improvement relative to the source compounds (averagely -0.89), as depicted in Fig. 3c. Additionally, it's limited to available compounds in the library without the ability to exploiting the whole chemical space, as shown in Fig. 3e. In deep learning models, Seq2seq has low valid rates (Fig. 3d) while G2G tends to generate outliers (Fig. 3e), resulting in an unsatisfactory success rate. The relatively high performance of the Seq2seq model indicates that the string-based models have the potential to design good hops if the issue of low success rate can be solved. Generally, only DeepHop can efficiently generate high-quality scaffold hops by a balanced performance in three measurements and producing similar distributions of chemical property to the source compounds, leading to significantly higher success rates and constraint success rates.

**Fig. 4:**
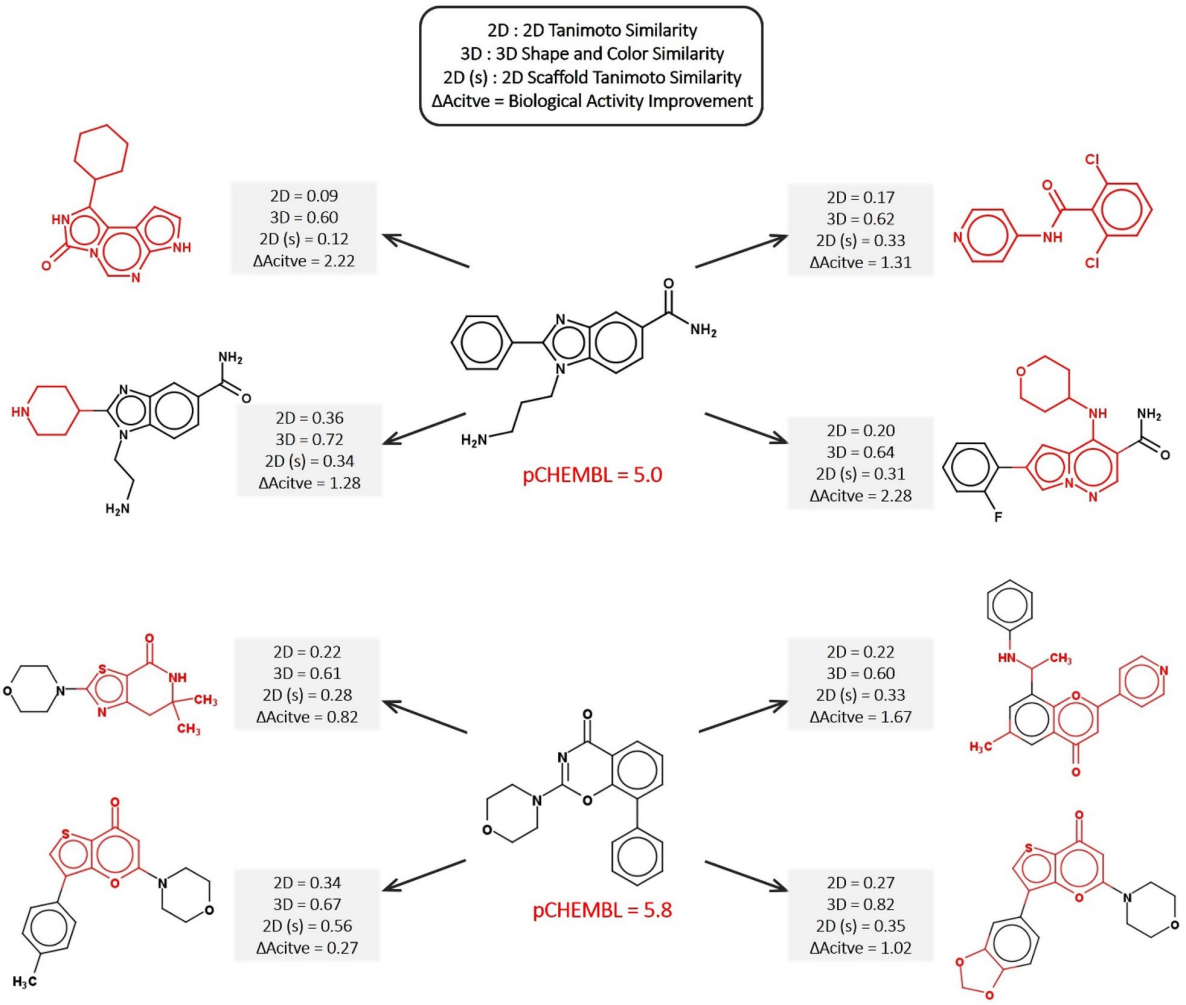
Example of top-4 successful hops with two test molecules generated by DeepHop for CHEMBL267. The changes in the generated molecules compared with starting molecule are highlighted in red

Figure 4 shows two examples of the top-predicted molecules generated by DeepHop. The modified groups lead to significant changes in 2D while small changes in 3D. More cases are shown in Additional file 1: Figs.S4, S5.

### Model performance on unseen targets

We have shown that DeepHop achieves good performance in the internal test set. However, in real-world cases, scaffold hopping is often required for target proteins that have only a few known active compounds, and thus it is unable to construct sufficient scaffold hopping pairs for training. To mimic this scenario, we further examined whether DeepHop can be generalized to external targets that have never been observed in the training set. Following the same sampling strategy as above, we generated ten molecules for each parent molecule on six unseen targets.

As shown in Table 3, the homogeneous targets performed very well in the external test set, even if all the molecular structures and protein sequences in these tasks have never been observed by the model. The results are expected as the deep learning models are often capable of generalizing similar tasks. It also suggests that when there are only a few known actives for a specific target protein that has over 60% sequence identity similarity to the training target proteins, DeepHop can be alternatively applied to generate scaffold hops directly without the need of re-training from scratch.

**Table 3:** The independent tests of three heterogeneous proteins without homologs and three homogeneous proteins with homologs to the proteins in the training set

Metrics	ChEMBL Target					
	Homologs			Non-homologs		
	CHEMBL 4225	CHEMBL 2041	CHEMBL 2292	CHEMBL 2208	CHEMBL 4523	CHEMBL 2147
Success Rate	0.765	0.630	0.705	0.024	0.055	0.129
Constraint Success	0.471	0.519	0.341	0.024	0.009	0.036
Improvement	0.515	1.259	0.824	− 0.378	− 1.210	− 1.263

As expected, the model achieved low success rates on three heterogeneous protein targets that are non-homologous to our training proteins (sequence ID < 25%). The low rates were mostly caused by the drop in bioactivity improvement. For the heterogeneous target proteins, we wonder how many scaffold pairs are required to achieve a decent hopping. To this end, we equipped the model with the scheme of transfer learning and tested how well it can design inhibitors for unfamiliar proteins. Specifically, the trained DeepHop were fine-tuned with 5, 20, 50, 80% of scaffold hopping pairs from each unseen target protein, respectively.

**Fig. 5:**
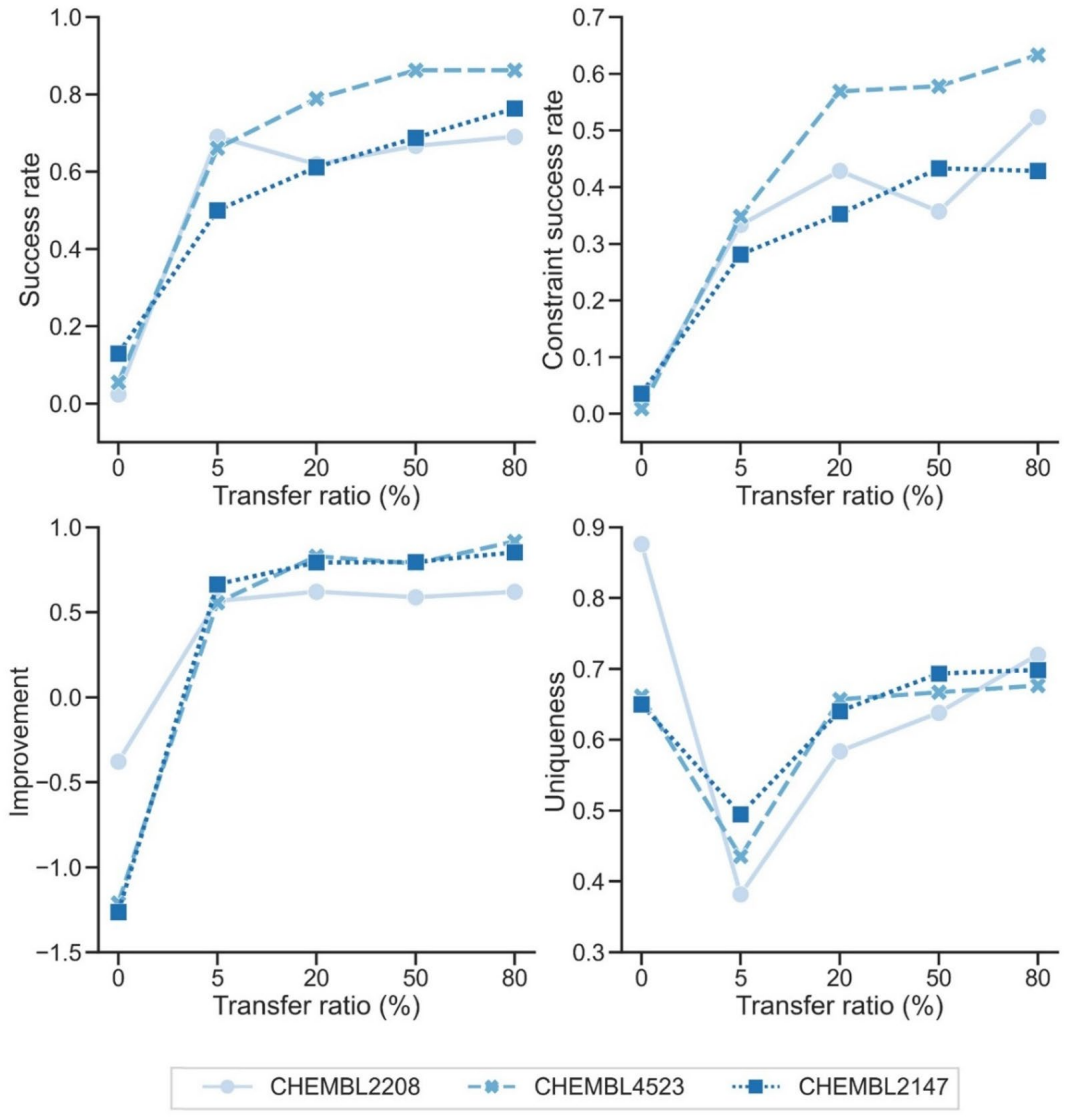
Transfer learning with different ratios of scaffold hopping pairs on the heterogeneous unseen protein targets

As shown in Fig. 5, with transfer learning, only 5% (around 40 ~ 200, see more details in Additional file 1: Table S8–S12) scaffold pairs can help unseen proteins to achieve fair success rates. At this point, the uniqueness of the generated molecules is poor because of the overfitting of limited data points. Thereafter, with the increase of scaffold hopping pairs, the model can gradually achieve a decent level of success rates and uniqueness. Note that the improvements are stable after fine-tuning 5% pairs, suggesting that the bioactivity feature is easy to capture compared to structural ones. These results demonstrate that DeepHop can be further generalized to non-homologs proteins with few-shot active compounds.

### Scaffold hopping case study

Next, we chose PIM-1 kinase (CHEMBL2147), a well-studied target for antitumor drugs, as a representative to mimic a real-world scaffold hopping process. To search for novel inhibitors of the PIM-1 kinase, Saluste and co-workers once reported a typical fragment hopping by replacing imidazopyridazine scaffold with triazolopyridine, which maintained the primary activity and significantly improved off-target selectivity as well as ADME property.

We started with one lead inhibitor (seed 1, IC_50_ = 0.024 nM) and two hit inhibitors (seed 2, IC_50_ = 155 nM; seed 3, IC_50_ = 130 nM), and aimed to generate potential scaffold hopping candidates with the improved pharmaceutical property. We used the trained model to generate 500 candidates for three seed compounds, respectively. All the generated candidates were then carried out with the docking process using AutoDock Vina [67].

As shown in Table 4, DeepHops can generate a large number of novel hops for each molecule by simply increasing the beam search width. The uniqueness values for seeds 1–3 are 77.4%, 52.8% and 66.4%, respectively. Among them, there are 51, 66, and 40 structurally successful hops generated for seed 1,2, and 3, meeting the requirements of (2D scaffold similarity ≤ 0.6)$\cap$(3D similarity (X; Y) ≥ 0.6). In terms of bioactivity, we found that 26.4%, 69.7%, and 60.8% of generated hops have a better docking score than the seed compounds, demonstrating the effectiveness of our model. It is worth noting that even though seed 1 has extremely high activity (IC_50_ = 0.024 nM), there are 11 molecules to have better-predicted activities and 102 molecules that have better docking scores, suggesting that DeepHop could be a powerful tool in developing Me-too or Me-better molecules.

**Table 4:** Scaffold hopping case study on PIM-1 kinase with three seed compounds

Metrics	Scaffold Hopping		
	Seed 1	Seed 2	Seed 3
Unique structures	387	264	332
Structurally successful hops	51	66	40
Predicted activity < Lead	11	138	167
Docking score < Lead	102	184	202

**Fig. 6:**
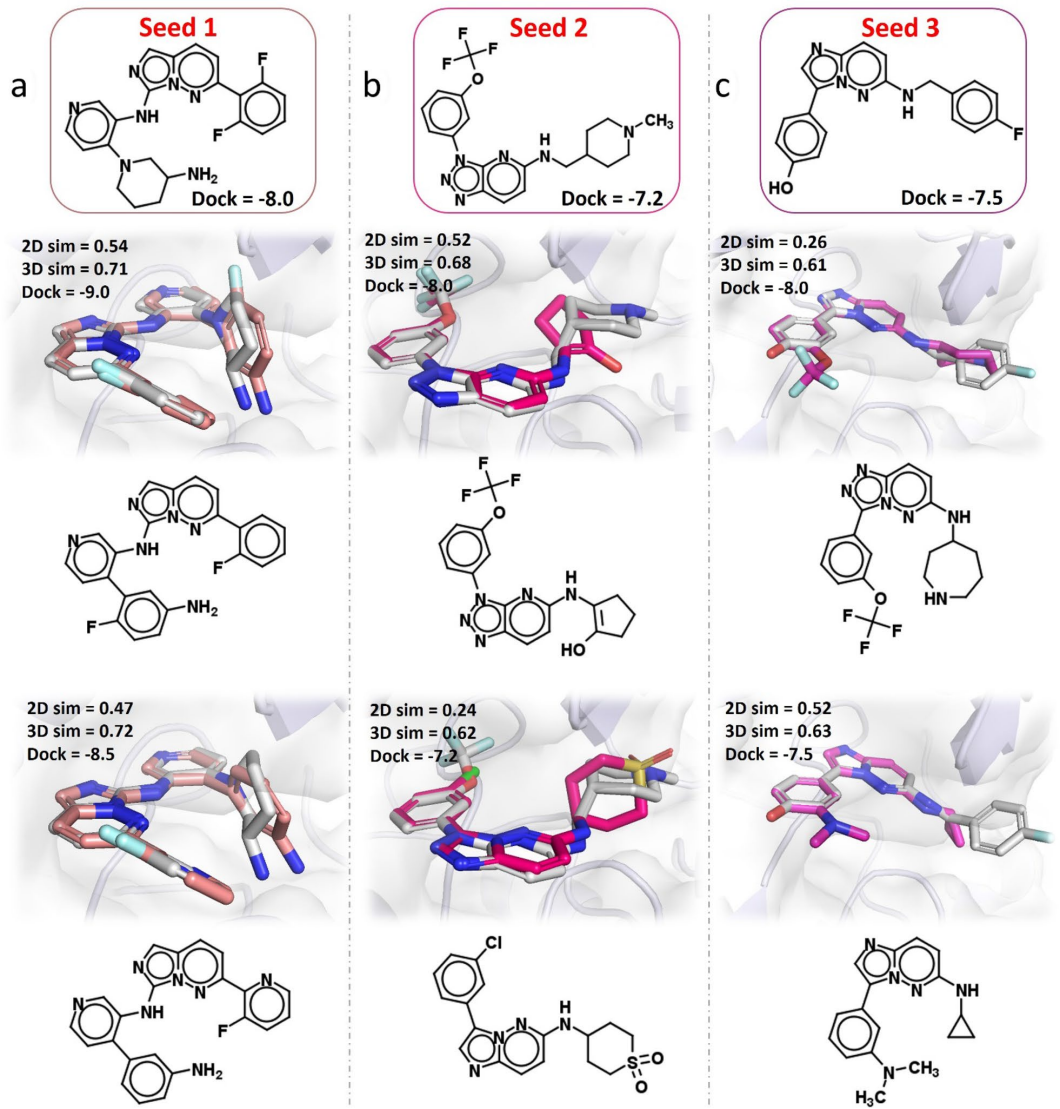
Overlay of the seed inhibitors (sliver) and top-predicted hops (colors). The 2D structures are shown below, and the structural similarity (2D Scaffold Tanimoto Similarity and 3D Shape and Color Similarity) and docking scores (kcal/mol) are attached in the upper left. Protein structure is retrieved from 5KZI [68]

Several examples are shown in Fig. 6. All scaffold hops meet the condition of the structure while obtaining similar or improved activities compared to the starting seeds.

## Discussions and conclusion

In this study, we have proposed a novel multimodal deep generative model, DeepHop, for scaffold hopping, which is a critical task in rational drug design. The model can generate large sets of potential hops with novel backbones and improved bioactivities. This can be used in not only early drug discovery phases like hit-to-lead or lead optimization but also patent busting for Me-Too and Me-Better molecules. Furthermore, we demonstrated that the model could generalize to new target proteins if fine-tuning with a small set of active compounds. This enables the generation of scaffold hops in low source scenarios. Through several case examples, we have shown that our method can be applied to practical scaffold hopping tasks, where most of the generated molecules have better docking scores than the original seeds while maintaining 3D similar but 2D dissimilar structure.

We see three main advantages of our works. First, it provides an entirely data-driven scaffold hopping strategy. The Transformer model implicitly learns the chemical hopping rules and performs candidate ranking via the beam search decoding procedure, without any pre-definition of screening databases and hand-encoded rules. Second, the DeepHop is easy to train and to use, and can be adapted to different datasets without any modifications to the model architecture. Furthermore, the DeepHop scales better to larger training data sets, different from rule-based expert systems that need to define rules in the knowledge base manually and are hard to process large training datasets.

There are also several weak points in our model. One major problem is diversity. The diversity of the target chemical space is constrained by the limited high bioactive compounds. It can be alleviated by active learning and iterative learning. Secondly, the evaluation of scaffold hopping should also be re-considered. The definition of what constitutes a scaffold hop is highly subjective and often differs, and currently, there is no accepted metric available for the evaluation of the scaffold hopping potential. Li et al. [69] once introduced a mathematical function to quantify the "chemical distance" between scaffolds, which seems to be a rigid metric. To further advance research activities directed at scaffold hopping and to make the performance of different methods comparable, there is a need to establish generally applicable scaffold definitions and retrieval metrics.

We also noted the model couldn't fully utilize protein information, especially protein 3D structural information. Although the inclusion of protein sequence enables successful hopping for homologous proteins, it can't recognize the complex protein-drug interactions. Fortunately, we have shown this can be solved by transferred learning over dozens of known active compounds. In the future, we may include a pre-trained protein-drug interaction network for a more accurate prediction of protein-drug interactions.

In addition, the definition of scaffold hopping in our work can be treated as a conditioned topological transformation, which is also defined as 4°hopping [9]. An obvious direction for further exploration is to classify the types of scaffold hopping by analyzing the molecular transformation paradigm. The hopping mode, like heterocycle replacement, ring-opening, and ring closure should become a controlled condition that guide the scaffold hopping mode. Another interesting extension to the DeepHop models would be to use multi-objective reinforcement learning to allow our generated hops to match the comprehensive expectation (e.g., scaffold replacement, ADMET, synthesizability) of medicinal chemists [35].

In summary, DeepHop provides a novel method that can perform target-based scaffold hopping and can generalize to new target proteins through fine-tuning with a small set of active compounds.We believe that the strategies described in our work will inpire future hit optimization works.

## Supplementary Information

**Additional file 1: Table S1. **3D GNN’s initial atom features calculated using RDKit. **Table S2. **Key hyperparameters, parameters for the best model in bold. **Table S3. **Details of selected targets in internal datasets. **Table S4.** Performance comparison of models on the Tyrosine-protein kinase JAK1 (CHEMBL2835). **Table S5.** Performance comparison of models on the Tyrosine-protein kinase SRC (CHEMBL267). **Table S6.** Performance comparison of models on the PI3-kinase p110-gamma subunit (CHEMBL3267). **Table S7.** Performance comparison of models on the Ribosomal protein S6 kinase 1 (CHEMBL4501). **Table S8. **Details of selected targets in external datasets. Training Pairs Amount is the total amount of scaffold hopping pairs in the training sets per target. R^2^ and RMSE are the performance of their virtual profiling models. **Table S9.** The independent tests by DeepHop-noGNN. **Table S10.** The independent tests by DeepHop-noProtein. **Figure S1. **The RMSE results of four models over 40 target proteins. **Figure S2. **The R^2^ results of four models over 40 target proteins. **Figure S3. **Performance of DeepHop model on the scaffold hopping datasets over 40 kinases protein compared to the DeepHop-noGraph and DeepHop-noProtein. **Table S11. **Key hyperparameters for the seq2seq model. **Table S12. **Key hyperparameters for the G2G model. **Figure S4.** Example of top-4 successful hops ((2D scaffold similarity ≤ 0.6)∩(3D similarity (X; Y) ≥ 0.6)∩Activity Improvement ≥ 1 ) with three test molecules generated by DeepHop for CHEMBL267. **Figure S5.** Example of top-4 successful hops ((2D scaffold similarity ≤ 0.6)∩(3D similarity (X; Y) ≥ 0.6)∩Activity Improvement ≥ 1 ) with three test molecules generated by DeepHop for CHEMBL267.

## Data Availability

Demo, instructions, and codes for DeepHop are available at https://github.com/prokia/deepHops.
