# Supplementary material for: Deep scaffold hopping with multimodal transformer neural networks
Source: J Cheminform. 2021 Nov 13;13:87. doi: 10.1186/s13321-021-00565-5 (PMC8590293; doi:10.1186/s13321-021-00565-5)
Supplement: Supplementary file 1 — Additional file 1: Table S1. 3D GNN’s initial atom features calculated using RDKit. Table S2. Key hyperparameters, parameters for the best model in bold. Table S3. Details of selected targets in internal datasets. Table S4. Performance comparison of models on the Tyrosine-protein kinase JAK1 (CHEMBL2835). Table S5. Performance comparison of models on the Tyrosine-protein kinase SRC (CHEMBL267). Table S6. Performance comparison of models on the PI3-kinase p110-gamma subunit (CHEMBL3267). Table S7. Performance comparison of models on the Ribosomal protein S6 kinase 1 (CHEMBL4501). Table S8. Details of selected targets in external datasets. Training Pairs Amount is the total amount of scaffold hopping pairs in the training sets per target. R2 and RMSE are the performance of their virtual profiling models. Table S9. The independent tests by DeepHop-noGNN. Table S10. The independent tests by DeepHop-noProtein. Figure S1. The RMSE results of four models over 40 target proteins. Figure S2. The R2 results of four models over 40 target proteins. Figure S3. Performance of DeepHop model on the scaffold hopping datasets over 40 kinases protein compared to the DeepHop-noGraph and DeepHop-noProtein. Table S11. Key hyperparameters for the seq2seq model. Table S12. Key hyperparameters for the G2G model. Figure S4. Example of top-4 successful hops ((2D scaffold similarity ≤ 0.6)∩(3D similarity (X; Y) ≥ 0.6)∩Activity Improvement ≥ 1 ) with three test molecules generated by DeepHop for CHEMBL267. Figure S5. Example of top-4 successful hops ((2D scaffold similarity ≤ 0.6)∩(3D similarity (X; Y) ≥ 0.6)∩Activity Improvement ≥ 1 ) with three test molecules generated by DeepHop for CHEMBL267. [file 13321_2021_565_MOESM1_ESM.docx]

**Additional file 1**

**Deep Scaffold Hopping with Multi-modal Transformer Neural Networks**

Shuangjia Zheng^1#^, Zengrong Lei^2#^, Haitao Ai^2^, Hongming Chen^3^, Daiguo Deng^2^*, Yuedong Yang^1^*

^1^School of Data and Computer Science, Sun Yat-sen University, China, 132 East Circle at University City, Guangzhou 510006, China

^2^Fermion Technology Co., Ltd, 1088 Newport East Road, Guangzhou 510335, China

^3^Centre of Chemistry and Chemical Biology, Guangzhou Regenerative Medicine and Health Guangdong Laboratory, Guangzhou 510530, China

Corresponding Authors

*Email: yangyd25@mail.sysu.edu.cn (Y.Y).

*Email: deco@fulmz.com (D.D).

**Table S1.** 3D GNN’s initial atom features calculated using RDKit.

| **Feature** | **Description** | **Size** |
| --- | --- | --- |
| Atom type | Atom type | 15 |
| Degree | The number of heavy atom neighbors | 7 |
| Number of hydrogens | The number of neighboring hydrogens | 5 |
| Formal charge | Atomic formal charge | 7 |
| Chirality | Unspecified, tetrahedral CW/CCW, or other | 4 |
| Hybridization | sp, sp*2*, sp*3*, sp*3*d, or sp*3*d*2* | 5 |
| Aromaticity | Whether atom is part of an aromatic system. | 1 |

**T****able S2.** Key hyperparameters, parameters for the best model in bold.

| Parameter | Values |
| --- | --- |
| Batch size | 2048, 4096, **8192** |
| Number of layers | 2, 4, **6**, 8 |
| Hidden states | 128, **256**, 512 |
| Attention heads | 2, 4, 6, **8** |
| Label smoothing | 0 ~ 1 (**0**) |
| Optimizer | SGD, **Adam,** Adagrad |
| Starting learning rate | 0.001 ~ 2 (**1)** |
| Decay factor | 0 ~ 1 (**0.5)** |
| Warming steps | 4000~8000 **(4750)** |
| Drop out | 0 ~ 1 **(0.1)** |

**Table S3.** Details of selected targets in internal datasets. Molecule Amount denotes the total amount of bioactivity instances in each target. Training Pairs Amount is the total amount of scaffold hopping pairs in the training sets per target. R^2^ and RMSE are the performance of their virtual profiling models.

| Metrics | ChEMBL Target | | | | |
| --- | --- | --- | --- | --- | --- |
|  | CHEMBL267 (Tyrosine-protein kinase SRC) | CHEMBL2835 (Tyrosine-protein kinase JAK1) | CHEMBL3267 (PI3-kinase p110-gamma subunit) | CHEMBL4501 (Ribosomal protein S6 kinase 1) |  |
| Abvs. | SRC | JAK1 | PI3G | S6K1 |  |
| Molecule Amount | 3172 | 3764 | 1913 | 1787 |  |
| Training Pairs Amount | 4281 | 5309 | 2631 | 2305 |  |
| R^2^ | 0.74 | 0.72 | 0.70 | 0.73 |  |
| RMSE | 0.67 | 0.60 | 0.60 | 0.61 |  |

**Table S4.** Performance comparison of models on the Tyrosine-protein kinase JAK1 (CHEMBL2835).

| Models | Metrics | | | | | |
| --- | --- | --- | --- | --- | --- | --- |
|  | **Validity (%)** | **Success rate (%)** | **Constraint**  **success rate**  **(%)** | **Improvement** | **Uniqueness (%)** |  |
| LBVS | **100** | 10.2 | 1.5 | -1.82 | **100** |  |
| MMPA | **100** | 15.7 | 6.6 | 0.29 | **98.6** |  |
| Seq2seq | 34.6 | 32.6 | 13.4 | 0.28 | 16.9 |  |
| G2G | 99.5 | 27.7 | 13.1 | **1.11** | 13.9 |  |
| DeepHop | 95.8 | **73.2** | **35.4** | 0.41 | 66.2 |  |

**Table S5.** Performance comparison of models on the Tyrosine-protein kinase SRC (CHEMBL267).

| Models | Metrics | | | | | |
| --- | --- | --- | --- | --- | --- | --- |
|  | **Validity (%)** | **Success rate (%)** | **Constraint**  **success rate**  **(%)** | **Improvement** | **Uniqueness (%)** |  |
| LBVS | **100** | 40.1 | 7.8 | -0.89 | **100** |  |
| MMPA | 91.1 | 32.9 | 15.8 | 0.47 | **100** |  |
| Seq2seq | 32.9 | 38.3 | 23.0 | **1.39** | 18.9 |  |
| G2G | 99.4 | 32.3 | 16.1 | 0.32 | 16.4 |  |
| DeepHop | 91.8 | **68.8** | **44.9** | 0.94 | 66.6 |  |

**Table S6.** Performance comparison of models on the PI3-kinase p110-gamma subunit (CHEMBL3267).

| Models | Metrics | | | | | |
| --- | --- | --- | --- | --- | --- | --- |
|  | **Validity (%)** | **Success rate (%)** | **Constraint**  **success rate**  **(%)** | **Improvement** | **Uniqueness (%)** |  |
| LBVS | **100** | 43.8 | 17.5 | -0.47 | **100** |  |
| MMPA | **100** | 45.6 | 10.5 | 0.11 | **100** |  |
| Seq2seq | 36.6 | 33.9 | 16.1 | 0.61 | 21.1 |  |
| G2G | 100 | 43.9 | 15.8 | 0.37 | 38.6 |  |
| DeepHop | 95.0 | **71.9** | **29.8** | **0.71** | 75.6 |  |

**Table S7.** Performance comparison of models on the Ribosomal protein S6 kinase 1 (CHEMBL4501).

| Models | Metrics | | | | | |
| --- | --- | --- | --- | --- | --- | --- |
|  | **Validity (%)** | **Success rate (%)** | **Constraint**  **success rate**  **(%)** | **Improvement** | **Uniqueness (%)** |  |
| LBVS | **100** | 44.4 | 14.8 | -0.60 | **100** |  |
| MMPA | **98.1** | 41.5 | 20.8 | 0.36 | **100** |  |
| Seq2seq | 12.8 | 11.8 | 3.9 | 0.79 | 14.5 |  |
| G2G | 100 | 29.6 | 13.9 | **1.70** | 2.6 |  |
| DeepHop | 92.8 | **46.3** | **24.1** | 1.16 | 74.5 |  |

**Table S8.** Details of selected targets in external datasets. Training Pairs Amount is the total amount of scaffold hopping pairs in the training sets per target. R^2^ and RMSE are the performance of their virtual profiling models.

| **Metrics** | **ChEMBL Target** | | | | | |
| --- | --- | --- | --- | --- | --- | --- |
|  | Homologs | | | Non-Homologs | | |
|  | CHEMBL 4225  (Dual specificity protein kinase CLK2) | CHEMBL 2041  (Tyrosine-protein kinase receptor RET) | CHEMBL 2292  (Tyrosine-phosphorylation kinase 1A) | CHEMBL 2208 (MAP activated kinase 2) | CHEMBL 4523 (Serine/threonine-protein kinase PIM2) | CHEMBL 2147 (Serine/threonine-protein kinase PIM1) |
| Training Pairs | 531 | 755 | 1109 | 859 | 2324 | 4363 |
| R^2^ | 0.67 | 0.66 | 0.67 | 0.69 | 0.69 | 0.69 |
| RMSE | 0.51 | 0.73 | 0.57 | 0.60 | 0.54 | 0.59 |

**Table S9.** The independent tests by DeepHop-noGNN.

| **Metrics** | **ChEMBL Target** | | | | | |
| --- | --- | --- | --- | --- | --- | --- |
|  | Homologs | | | Non-homologs | | |
|  | CHEMBL 4225 | CHEMBL 2041 | CHEMBL 2292 | CHEMBL 2208 | CHEMBL 4523 | CHEMBL 2147 |
| Success Rate | 0.664 | 0.528 | 0.683 | 0.012 | 0.067 | 0.094 |
| Constraint Success | 0.381 | 0.425 | 0.322 | 0.012 | 0.011 | 0.036 |
| Improvement | 0.409 | 1.012 | 0.807 | -0.152 | -0.988 | -1.518 |

**Table S10.** The independent tests by DeepHop-noProtein.

| **Metrics** | **ChEMBL Target** | | | | | |
| --- | --- | --- | --- | --- | --- | --- |
|  | Homologs | | | Non-homologs | | |
|  | CHEMBL 4225 | CHEMBL 2041 | CHEMBL 2292 | CHEMBL 2208 | CHEMBL 4523 | CHEMBL 2147 |
| Success Rate | 0.325 | 0.241 | 0.292 | 0.048 | 0.109 | 0.054 |
| Constraint Success | 0.201 | 0.153 | 0.182 | 0.036 | 0.088 | 0.028 |
| Improvement | 0.283 | 0.521 | 0.244 | -0.518 | -1.105 | -0.674 |

**Details of deep QSAR models’ architecture.**

**DMPNN-multi-task.** We constructed a multitask regression directed graph neural networks for the kinase bioactivity prediction following the implementation of Yang’s work^1^. DMPNN is a kind of message-passing graph neural network that passing messages with directed edges (bonds) rather than those with vertices. It has been shown promising results in molecular property predictions^1^.

**Table S9.** Key hyperparameters, parameters for the best model in bold.

| Parameter | Values |
| --- | --- |
| Batch size | 32, 64, **128** |
| Number of GNN layers | 2, **3**, 4 |
| Hidden states | 128, **256**, 512 |
| Optimizer | SGD, **Adam,** Adagrad |
| Learning rate | 0.001 |
| Drop out | 0 ~ 1 **(0.1)** |

**DNN-multi-task.** Following Xiong’s work^2^, we implemented a multitask regression DNN architecture with shared hidden layers across all tasks, where each task represents the bioactivity to be predicted against a specific kinase. Extended connectivity fingerprints (ECFP) of radius 2 with 1024 bits were adopted to featurize each molecule and fed into the input layer. It has been shown state-of-the-art results in virtual kinome-wide polypharmacology profiling^1^.

**Table S10.** Key hyperparameters, parameters for the best model in bold.

| Parameter | Values |
| --- | --- |
| Batch size | 32, 64, **128** |
| Number of layers | 2, **3**, 4, 5 |
| Hidden states | 512, **1024**, 2048 |
| Optimizer | SGD, **Adam,** Adagrad |
| Starting learning rate | 0.0001, 0.0005, **0.001** |
| Decay factor | 0 ~ 1 (**0.002)** |
| Drop out | 0 ~ 1 **(0.5)** |

We re-implemented these two models with Pytorch (version 0.4.0) and Tensorflow (version 1.6.0), respectively. All trainings are performed on standard NVIDIA GPUs. The code is developed in Python 3.6. For comparison, we also reported the results of the two models using the single-task training strategy (referred to as DMPNN-single and DNN-single).


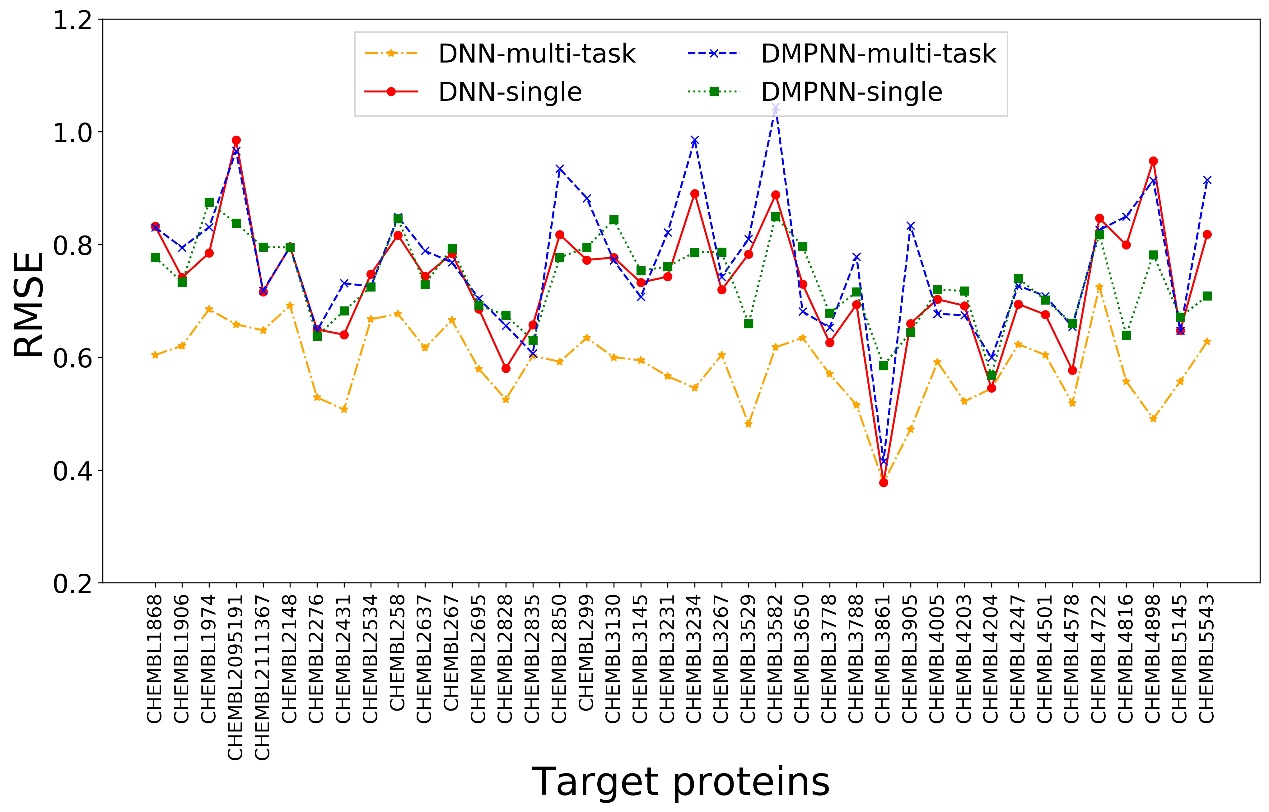


Figure S1. The RMSE results of four models over 40 target proteins. The DMPNN model was based on Yang’s work^1^ and the DNN-multi-task was based on Xiong’s work^2^. For each modeling method we used single-task and multi-task settings, respectively. We finally used DNN-multi-task as the virtual profiling models.


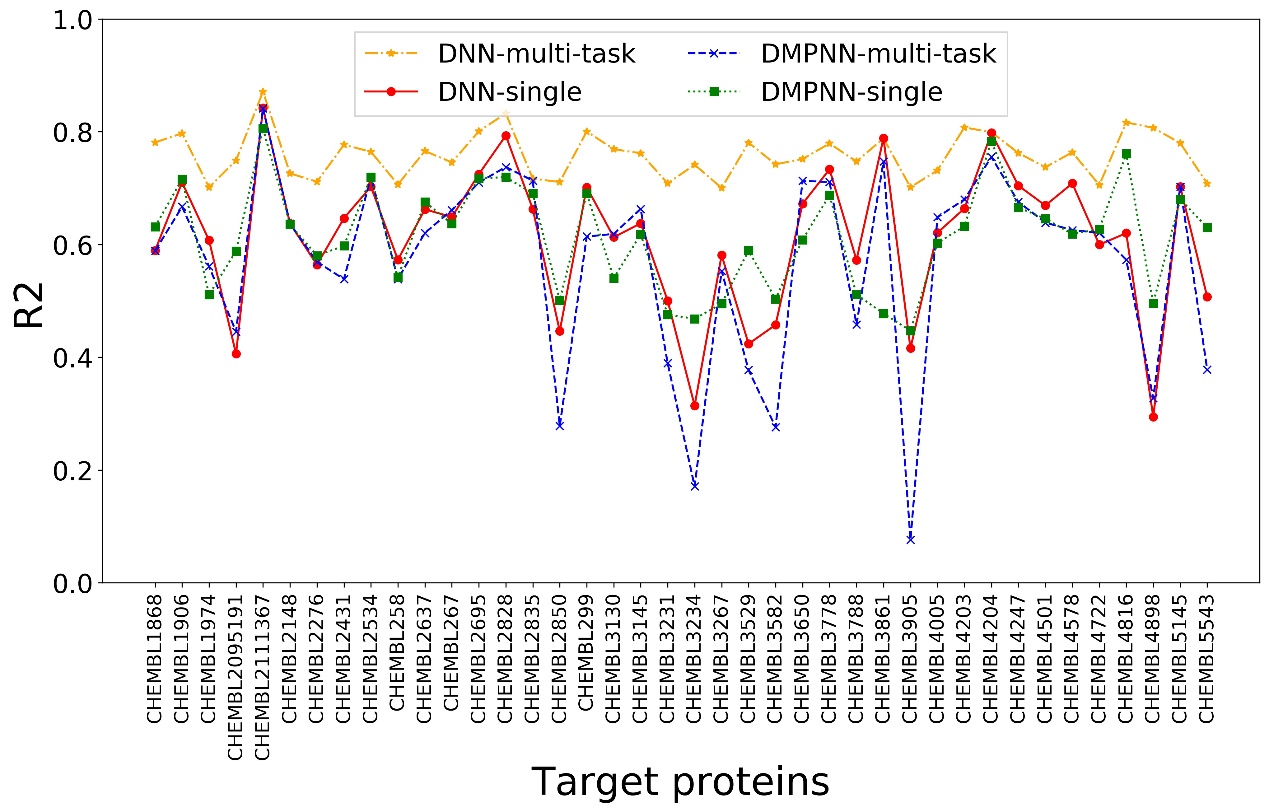


Figure S2. The R^2^ results of four models over 40 target proteins.


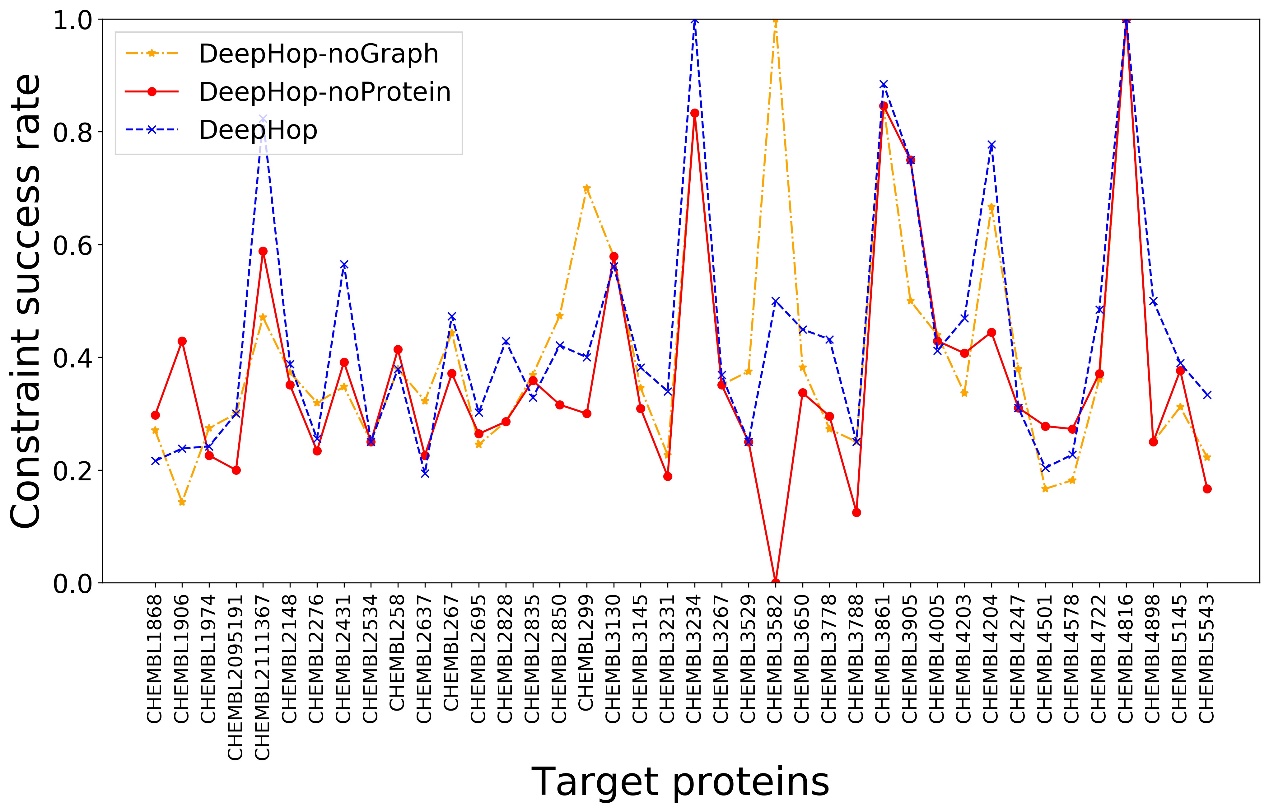


Figure S3. Performance of DeepHop model on the scaffold hopping datasets over 40 kinases protein compared to the DeepHop-noGraph and DeepHop-noProtein.

**Table S11.** Key hyperparameters for the seq2seq model.

| Parameter | Values |
| --- | --- |
| Batch size | 32 |
| Optimizer | Adam |
| Starting learning rate | 0.0001 |
| Max gradient norm | 5.0 |
| Attention dim | 512 |
| layers | 4 |
| Embedding dim | 512 |
| Max sequence length | 140 |
| Drop out | 0.8 |

**Table S12.** Key hyperparameters for the G2G model.

| Parameter | Values |
| --- | --- |
| Batch size | 32 |
| Optimizer | Adam |
| Starting learning rate | 0.001 |
| Message passing layer | 6 |
| Embedding dim | 300 |
| Graph decoder layer | 3 |
| Latent code dimension | 8 |


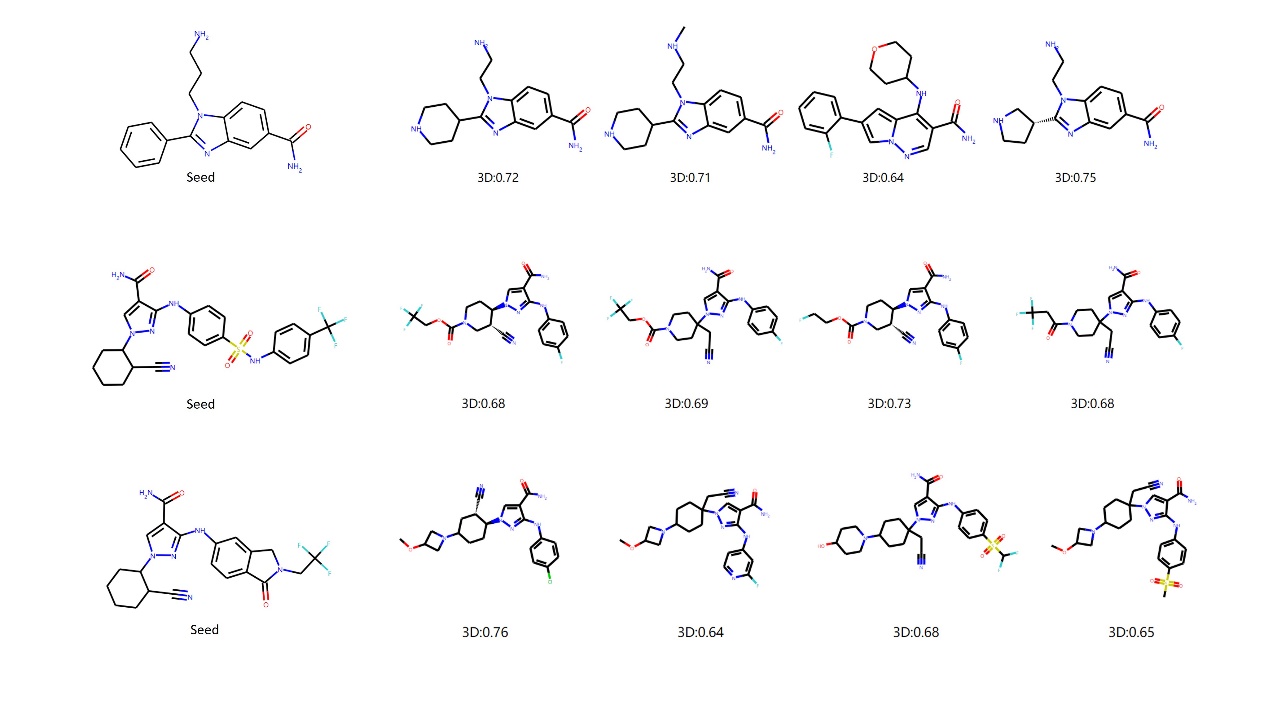


Figure S4. Example of top-4 successful hops ((2D scaffold similarity ≤ 0.6)∩(3D similarity (X; Y) ≥ 0.6)∩Activity Improvement ≥ 1 ) with three test molecules generated by DeepHop for CHEMBL267.


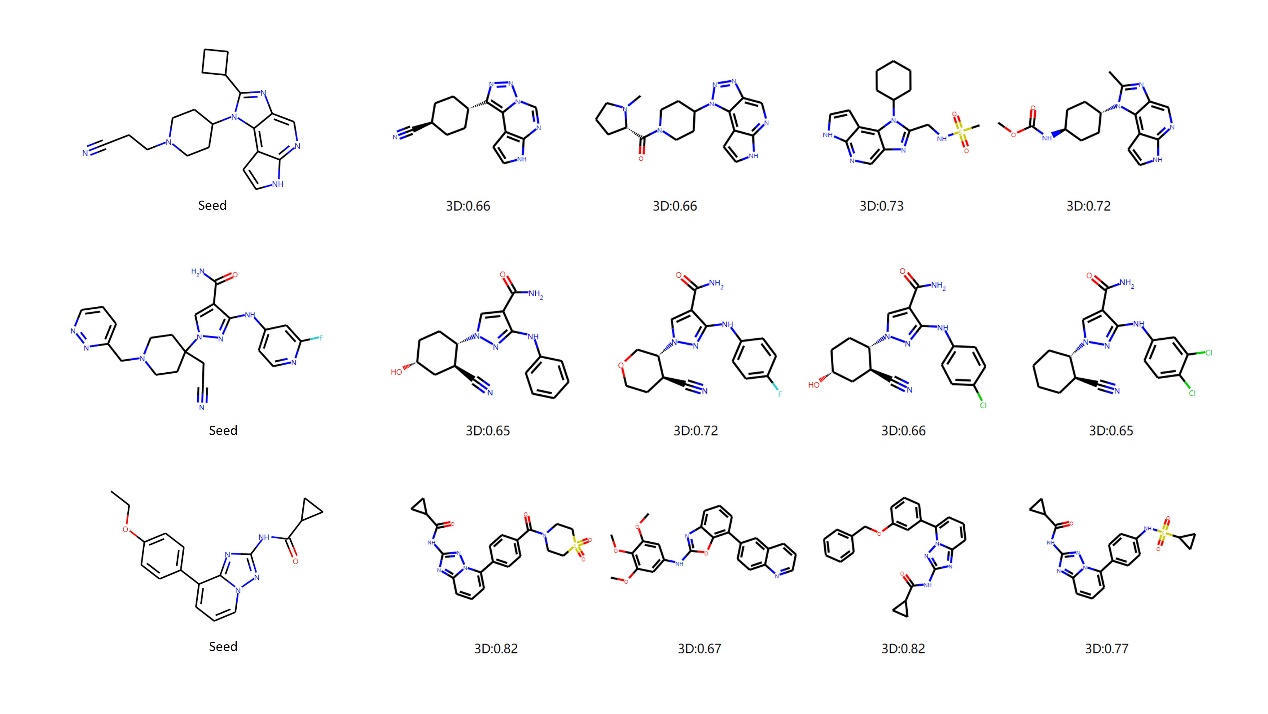


Figure S5. Example of top-4 successful hops ((2D scaffold similarity ≤ 0.6)∩(3D similarity (X; Y) ≥ 0.6)∩Activity Improvement ≥ 1 ) with three test molecules generated by DeepHop for CHEMBL267.

**Reference**

1. Yang, K.; Swanson, K.; Jin, W.; Coley, C.; Eiden, P.; Gao, H.; Guzman-Perez, A.; Hopper, T.; Kelley, B.; Mathea, M., Analyzing learned molecular representations for property prediction. *Journal of chemical information and modeling* **2019,** *59* (8), 3370-3388.

2. Li, X.; Li, Z.; Wu, X.; Xiong, Z.; Yang, T.; Fu, Z.; Liu, X.; Tan, X.; Zhong, F.; Wan, X.; Wang, D.; Ding, X.; Yang, R.; Hou, H.; Li, C.; Liu, H.; Chen, K.; Jiang, H.; Zheng, M., Deep Learning Enhancing Kinome-Wide Polypharmacology Profiling: Model Construction and Experiment Validation. *J Med Chem* **2020,** *63* (16), 8723-8737.
